# Simultaneous Determination of Six Active Compounds in Yixin Badiranjibuya Granules, a Traditional Chinese Medicine, by RP-HPLC-UV Method

**DOI:** 10.1155/2015/974039

**Published:** 2015-10-26

**Authors:** Ning Yu, ChenHui He, Gulistan Awuti, Cheng Zeng, JianGuo Xing, Wei Huang

**Affiliations:** ^1^College of Pharmacy, Shihezi University, Shihezi 832002, China; ^2^Xinjiang Institute of Metaria Medica, Urumqi 830004, China; ^3^Institute of Traditional Chinese Medicine, Xinjiang Medical University, Urumqi 830005, China; ^4^Department of Pharmaceutics, Institute of Materia Medica, Chinese Academy of Medical Sciences and Peking Union Medical College, Beijing 100052, China

## Abstract

In this study, a sensitive, precise, and accurate HPLC-UV method was developed and validated to simultaneously determine the six analytes (luteolin-7-*O*-*β*-D-glucuronide, apigenin-7-*O*-*β*-D-glucuronide, diosmetin-7-*O*-*β*-D-glucuronide, acacetin-7-*O*-*β*-D-glucuronide, tilianin, and rosmarinic acid) in Yixin Badiranjibuya Granules, in which five analytes (i.e., luteolin-7-*O*-*β*-D-glucuronide, apigenin-7-*O*-*β*-D-glucuronide, diosmetin-7-*O*-*β*-D-glucuronide, acacetin-7-*O*-*β*-D-glucuronide, and rosmarinic acid) were determined for the first time in Yixin Badiranjibuya Granules, the content of tilianin in Yixin Badiranjibuya Granules was reported in other literatures, and the content of tilianin in our work was higher than that of the literature reports. The quality of 11 batch samples from four different manufacturers was evaluated using the proposed determination method. The contents of the six analytes were largely different among samples from various manufacturers. Therefore, this determination method can provide a scientific basis for quality evaluation and control of Yixin Badiranjibuya Granules.

## 1. Introduction

Yixin Badiranjibuya Granule isa well-known traditional Chinese medicine officially listed in the Fascicle of Uygur Medicine of Drug Standard of Ministry of Public Health of the People's Republic of China. In clinical applications of traditional Chinese medicine, it has been proven effective in treating cardiovascular disease, fatigue insomnia, upset, asthma, and neurasthenia [1]. Modern pharmacology studies showed that the granules exert remarkable inhibitory effects on platelet aggregation in human blood; possess a tonic for heart, antiviral, antidiuretic, and antiasthmatic activities; suppress the generation of superoxide; and improve learning and memory abilities [2].

Yixin Badiranjibuya Granules are made of the aqueous extracts of* Dracocephalum moldavica L.* Previous studies demonstrated that the aqueous extracts of* D. moldavica* can be employed to treat cardiovascular diseases, promote vascular relaxation, and exert antioxidant activities [3–5].

**Figure 1:**
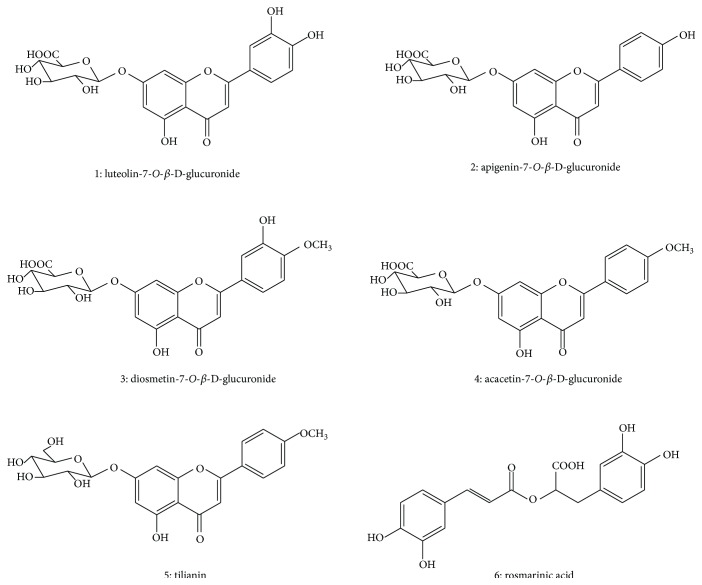
Chemical structures of six active compounds.

*D. moldavica* possesses key cardiac activities; it is used as a tranquilizer and remedy for relief of nervous conditions and for protecting cardiomyocytes against hypoxia/reoxygenation injury [6]. In addition, the water extracts of* D. moldavica* possess an anti-ischemic effect in animal experiments, act against isoproterenol-induced increase in myocardial oxygen consumption, and improve hypoxia tolerance [4, 7, 8]. Phytochemical studies on* D. moldavica* revealed that it contains many types of components, such as flavonoids, triterpenoids, steroids, phenylpropanoids, iridoids, and polysaccharides [9, 10]. More recently, our research group extensively investigated the chemistry of* D. moldavica*. Flavonoids, including luteolin-7-*O*-*β*-D-glucuronide (LTG), apigenin-7-*O*-*β*-D-glucuronide (AGG), diosmetin-7-*O*-*β*-D-glucuronide (DSG), acacetin-7-*O*-*β*-D-glucuronide (ACG), and tilianin (TLN), and phenylpropanoids, such as rosmarinic acid (RA), were identified as the major components of* D. moldavica* [11–13]. Their chemical structures are shown in Figure 1. Previous studies demonstrated that the total flavonoids extracted from* D. moldavica* possess obvious protective effects on myocardial I/R injury [14–16]. RA possesses a number of interesting biological activities, such as anti-inflammatory, antioxidant, antibacterial, immunoregulatory, and mesangial cell proliferation-inhibitory effects [17–20].

Herbal drugs contain a myriad of compounds in complex matrices in which no single active constituent is responsible for the overall efficacy. Although the application of Yixin Badiranjibuya Granules has been growing steadily in recent years, we have not found any scientific reports assessing the quality of these granules. Therefore, a quantitative method for evaluating the quality consistency of Yixin Badiranjibuya Granules should be developed. Consequently, a new RP-HPLC method for the simultaneous quantitative analysis of six active components (LTG, AGG, DSG, ACG, TLN, and RA) is the most direct and important method for the quality control of Yixin Badiranjibuya Granules.

## 2. Experimental

### 2.1. Reagents and Materials

Reference substance of RA (purity ≥ 98%) was purchased from the National Institutes for Food and Drug Control (Beijing, China). Reference substances of LTG and AGG (purity ≥ 98%) were purchased from Shanghai Forest Biotechnology Limited Company (Shanghai, China).

Reference substances of DSG, ACG, and TLN were separated and purified in Shanghai Institute of Pharmaceutical Industry, and the study method and data were published on Biochemical Systematics and Ecology magazine [12]. The purities of DSG, ACG, and TLN were determined to be more than 98% by HPLC analysis, and the purities and data of MS and NMR are shown in the appendix.

Acetonitrile and formic acid (HPLC grade) were purchased from Fisher Scientific Products (Fair Lawn, NJ, USA). Deionized water was prepared with a Millipore water purification system (Millipore, Milford, MA, USA) and then filtered through a 0.45 *μ*m micropore membrane. Analytical grade methanol (Tianjin Chemical, Tianjin, China) was used for sample preparation.

Eleven batches of Yixin Badiranjibuya Granule samples were purchased from four different manufacturers in China. These manufacturers were Xinjiang Uygur Pharmaceutical Co., Ltd. (A), Wuhu Luye Pharmaceutical Co., Ltd. (B), Shanxi Dongtai Pharmaceutical Co., Ltd. (C), and Wuhan Jianmin Pharmaceutical Group Limited by Share Ltd. (D). Samples 1–3 were produced from manufacturer A, samples 4–7 were produced from manufacturer B, samples 8 and 9 were produced from manufacturer C, and samples 10 and 11 were produced from manufacturer D.

### 2.2. Instruments

Liquid chromatographic analyses were performed using a Shimadzu system that comprised LC-20AT pumps and SPD 20A UV-visible detector connected to Shimadzu Spin Chrome software.

### 2.3. Liquid Chromatographic Conditions

For the quantification of LTG, AGG, DSG, ACG, TLN, and RA, Merck Purospher STAR C_18_ column (250 mm × 4.6 mm, 5 *μ*m) was used to separate the six active compounds. Elution was achieved at 35°C with a multilinear gradient of A (water with 0.5% formic acid) and B (acetonitrile) under the following conditions: 0–30 min, isocratic 18% (v/v) B; 30–60 min, linear gradient 18%–30% (v/v) B; and 60–70 min, isocratic 30% (v/v) B. The flow rate was 1.0 mL/min. The UV spectra were recorded between 190 and 370 nm, and a wavelength of 330 nm was chosen for recording. The injection volume was 10 *μ*L.

### 2.4. Sample Solutions Preparation

The extraction conditions of Yixin Badiranjibuya Granule samples were optimized as follows: Yixin Badiranjibuya Granule samples were ground to a powder and then passed through a 60-mesh sieve. Pulverized samples (3.0 g) were weighed accurately and then extracted via an ultrasonic bath (40 kHz, 250 W) using methanol-water (70 : 30, v/v) for 30 min at room temperature, and the ratio sample/extraction solvent is 3/100 (g/mL).

### 2.5. Preparation of Standard Solutions and Calibration Curves

Stock solutions of six reference substances were individually prepared by dissolving 3.98 mg of LTG, 2.91 mg of AGG, 3.84 mg of DSG, 5.85 mg of ACG, 9.88 mg of TLN, and 3.48 mg of RA in 25 mL of methanol-water (70 : 30, v/v). A certain volume of individual reference substance for these six reference substances was accurately weighed, stored in the same 10 mL volumetric flasks, and added with methanol-water (70 : 30, v/v) to dilute into mixed reference solutions with a series of concentrations. Standard solutions for LTG (3.184, 7.96, 15.92, 23.88, 27.064, and 31.84 *μ*g/mL), AGG (1.165, 3.495, 5.825, 11.65, 23.3, and 34.95 *μ*g/mL), DSG (2.784, 6.96, 13.92, 16.704, 22.272, and 27.84 *μ*g/mL), ACG (2.34, 7.02, 11.70, 23.40, 46.80, and 70.20 *μ*g/mL), TLN (7.9, 11.85, 19.75, 27.65, 31.60, and 39.50 *μ*g/mL), and RA (2.184, 6.552, 10.92, 15.228, 17.472, and 21.84 *μ*g/mL) were prepared and analyzed. The calibration curves were constructed by plotting the peak areas versus the concentrations of each analyte. The limits of detection (LOD) and quantification (LOQ) for each analyte were determined by serial dilution of standard solution until the signal-to-noise (*S*/*N*) ratios were 3 and 10, respectively.

### 2.6. Method Validation

The method was validated in terms of parameters of linearity, LOD and LOQ, precision, accuracy, stability, and repeatability according to the International Conference on Harmonisation (ICH) [21].

With established chromatographic conditions, intra- and interday assays were used to determine the precision of the developed assay. For intraday precision, the sample was extracted and analyzed for six replicates within 1 d. For interday precision, the sample was extracted and examined in duplicates for over six consecutive days. Precisions were expressed by the relative standard deviations (RSDs).

Accuracy was evaluated with recovery test. Recovery was used to evaluate the method's accuracy by spiking known amounts of the six analyte solutions mixed with a certain amount (1.5 g, 60 mesh) of sample at three concentration levels (low, middle, and high) with three parallel analyses at each level. These mixtures were extracted and analyzed using the aforementioned method. The accuracy was evaluated by calculating the mean recovery of the six analytes. The recovery was measured according to the following formula:

(1) recovery%=(amount detected − original amount)amount spiked×100%,RSD%=SDmean×100%,

and the results were estimated with the RSD.

The stability of the sample solution was tested at room temperature. The sample was extracted using the aforementioned method, and the sample solution was analyzed using the established method at 0, 2, 4, 6, 8, 10, and 12 h. The peak areas of six analytes were recorded, and the RSDs of peak areas at different times were calculated. In terms of repeatability, the independent sample solution from identical batches was prepared and analyzed in six parallel analyses.

## 3. Result and Discussion

### 3.1. Optimisation of Liquid Chromatographic Conditions

**Figure 2:**
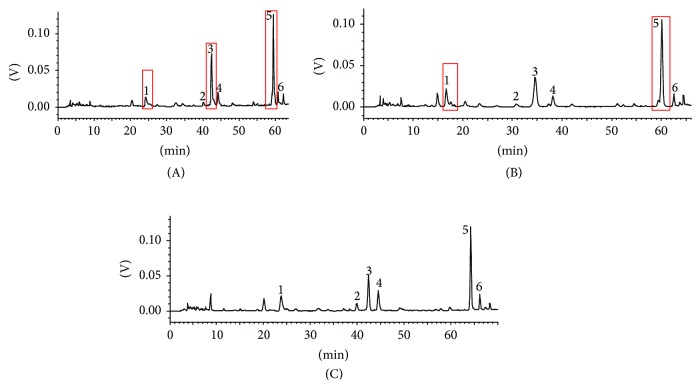
HPLC chromatograms of the sample solution under different conditions (1: luteolin-7-*O*-*β*-D-glucuronide; 2: apigenin-7-*O*-*β*-D-glucuronide; 3: diosmetin-7-*O*-*β*-D-glucuronide; 4: acacetin-7-*O*-*β*-D-glucuronide; 5: tilianin; 6: rosmarinic acid).

To successfully separate the six analytes, various analytical columns were tested, and different mobile phase compositions were changed to achieve good peak shape and high peak responses. First, the commonly used reversed-phase column, Shim-pack ODS C_18_ column, and Merck Purospher STAR C_18_ column with the same column length, diameter, and particle size (250 mm × 4.6 mm, 5 *μ*m) were compared using the same mobile phase program. The column temperature was 35°C. The mobile phase consisted of A (water with 0.5% formic acid) and B (acetonitrile), and the different separation systems are shown in Table 1. The elution programs were as follows: 0–30 min, isocratic 13% (v/v) B; 30–55 min, linear gradient 13%–22% (v/v) B; and 55–70 min, linear gradient 22%–40% (v/v) B. The flow rate was 1.0 mL/min, and the injection volume was 10 *μ*L. These analytes were monitored at 330 nm. Compounds 1, 3, and 5 could not be completely separated on Shim-pack ODS C_18_ column (Figure 2(A)). The results showed that the separation of the six analytes seemed better on the Merck Purospher STAR LP C_18_ column than that on the Shim-pack ODS C_18_ column. However, the efficiency of the separation of the peaks of compounds 1 and 5 was not very ideal on the Merck Purospher STAR LP C_18_ column (Figure 2(B)). Thus, the Merck Purospher STAR LP C_18_ column was selected to further optimize the ratio of the mobile phase program. When elution programs were as follows: 0–30 min, isocratic 18% (v/v) B; 30–60 min, linear gradient 18%–30% (v/v) B; and 60–70 min, isocratic 30% (v/v) B, the results showed that the separation performance of the six analytes was further improved (Figure 2(C)). Typical chromatograms of the sample solution are depicted in Figure 2.

### 3.2. Optimization of Extraction Conditions

**Figure 3:**
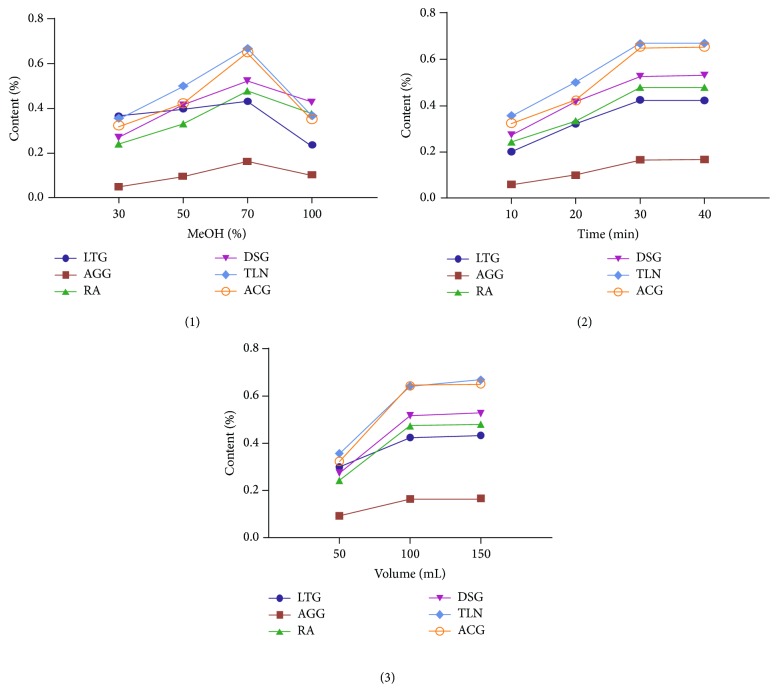
Optimization of extraction procedure.

Ultrasonic bath extraction is a sample extraction system for medicinal herbs, which has advantages of short operation time, high extraction efficiency, less solvent consumption, and good repeatability. The different ultrasonic bath extraction conditions of the six analytes were optimized using a univariate approach in terms of methanol concentration (30%, 50%, 70%, and 100% aqueous methanol), the ratio sample/extraction solvent (3/50, 3/100, and 3/150 g/mL), and extraction time (10, 20, 30, and 40 min), and the different ultrasonic bath extraction conditions are shown in Table 2. The individual experiments under different conditions were conducted in triplicate. The contents of the six analytes were calculated to evaluate the extraction efficiency, and the results are shown in Figure 3. For methanol concentration, the highest contents of the six analytes could be obtained at 70% aqueous methanol. For extraction time, the contents of the six analytes were significantly higher at 30 and 40 min than those at 10 and 20 min. For the ratio sample/extraction solvent, no significant differences were found for 3/100 and 3/150 (g/mL). To ensure a shorter time cost for the assay, the final conditions were optimized. Pulverized granules (3.0 g) were weighed accurately, and samples were extracted via ultrasonic bath (40 kHz, 250 W) using 70% aqueous methanol for 30 min at room temperature, and the ratio sample/extraction solvent is 3/100 (g/mL).

The working standard and sample solutions for analysis were ultimately obtained after filtering through a 0.45 *μ*m membrane filter, and 10 *μ*L was injected for HPLC analysis. All solutions were stored in the refrigerator at 4°C.

### 3.3. Method Validation

With the established chromatographic conditions, the results of the calibration curves, LODs, and LOQs are summarized in Table 3. These six standard compounds showed good linearity over a wide concentration range based on the correlation coefficients (*R* ^2^ > 0.9987). The LODs of the six analytes were in the range of 0.1165–0.3800 *μ*g/mL, and the LOQs of the six analytes were in the range of 0.3599–1.1508 *μ*g/mL. Only the content of TLN in Yixin Badiranjibuya Granules was reported in other literatures [22], and its LOD and LOQ are higher than our established method. So, these values indicated that the analytical method was sufficiently sensitive. The results of precision are shown in Table 4. The RSDs of the intra- and interday variations of the six analytes were less than 1.56% and 1.75%, respectively, indicating that the developed method was precise for the determination of these analytes. The results of repeatability and stability are shown in Table 5. In terms of repeatability, the independent sample solution from identical batches was prepared and analyzed in six parallel measurements (RSD < 1.80%). The sample solution was found to be stable. The RSDs of these peak areas were <1.87%. The recoveries of the six analytes are summarized in Table 6. The overall recoveries of the six analytes were 98.43%–101.09% with RSD < 1.81%, which suggested that this newly established method was highly accurate for the determination of the six analytes.

These data demonstrated that the established approach was precise, accurate, and sensitive for the simultaneous quantitative determination of the six analytes in Yixin Badiranjibuya Granules.

### 3.4. Quantification of the Six Analytes in Yixin Badiranjibuya Granules by HPLC

The newly established quantitative method was applied to evaluate the quality of 11 batch samples from four different manufacturers. Typical HPLC chromatograms of Yixin Badiranjibuya Granule samples are shown in Figure 3, and the contents of the six analytes are summarized in Table 7. As shown in Figure 3, the six analytes were the major components of Yixin Badiranjibuya Granules. All six analytes analyzed were detectable in the 11 batch samples of four different manufacturers. Table 7 also illustrates the significant differences in the content of single components of the six compounds among commercial samples from four different manufacturers. The content of the six compounds to be produced in Chinese Xinjiang Uygur Pharmaceutical Co., Ltd., was higher than that of the others. Thus, the newly established quantitative method could be used under similar chromatographic conditions to determine multiple main composition contents in Yixin Badiranjibuya Granules.

## 4. Conclusion

The chemical composition of traditional Chinese medicine is complex. Simultaneous determination of various active ingredients is necessary to ensure the quality of medicine and enhance drug quality control standards.

**Figure 4:**
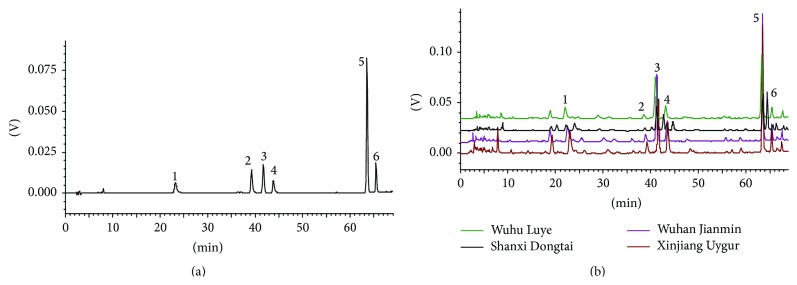
Typical HPLC chromatograms of Yixin Badiranjibuya Granule samples. (a) Mixed reference compounds. (b) Samples from four different manufacturers (1: luteolin-7-*O*-*β*-D-glucuronide; 2: apigenin-7-*O*-*β*-D-glucuronide; 3: diosmetin-7-*O*-*β*-D-glucuronide; 4: acacetin-7-*O*-*β*-D-glucuronide; 5: tilianin; 6: rosmarinic acid).

Flavonoids and phenylpropanoids are the main active ingredients in the traditional Chinese Uyghur medicine Yixin Badiranjibuya Granules. In this study, a sensitive, precise, and accurate HPLC-UV method was developed and validated to simultaneously determine the six analytes (five flavonoids and one phenylpropanoids) in Yixin Badiranjibuya Granules. The quality of 11 batch samples from four different manufacturers was evaluated using the proposed determination method (Figure 4). The contents of the six analytes were largely different among samples from various manufacturers, in which five analytes (i.e., luteolin-7-*O*-*β*-D-glucuronide, apigenin-7-*O*-*β*-D-glucuronide, diosmetin-7-*O*-*β*-D-glucuronide, acacetin-7-*O*-*β*-D-glucuronide, and rosmarinic acid) were determined for the first time in Yixin Badiranjibuya Granules, the content of tilianin in Yixin Badiranjibuya Granules was reported in other literature, and the content of tilianin in our work was higher than that of the literature reports [22]. Therefore, this determination method can provide a scientific basis for quality evaluation and control of Yixin Badiranjibuya Granules.

## Figures and Tables

**Figure 5:**
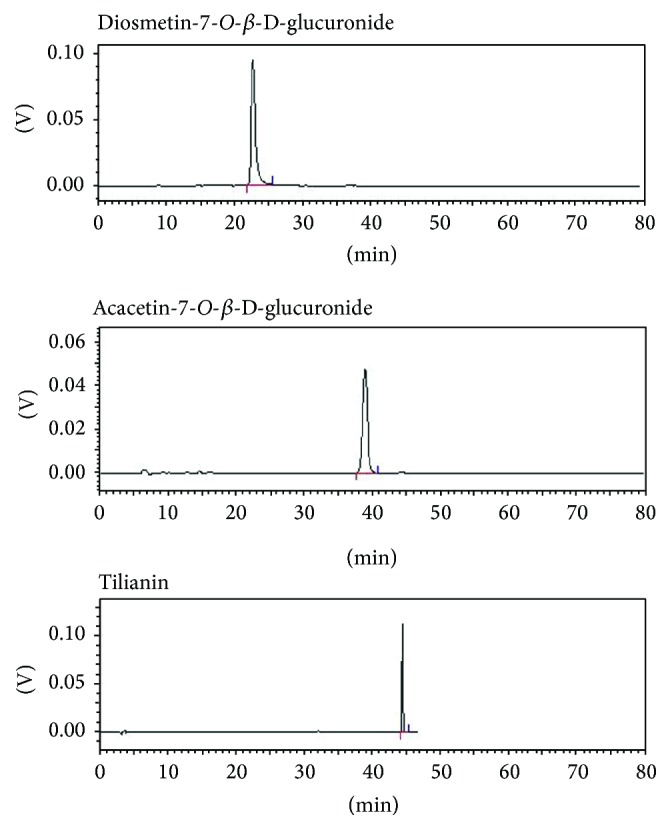
The purities of diosmetin-7-*O*-*β*-D-glucuronide, acacetin-7-*O*-*β*-D-glucuronide, and tilianin.

**Table 1:** The different separation system (see Figure 2).

Chromatograms	Column	Elution programs
(A)	Shim-pack ODS C_18_ column	0–30 min, isocratic 13% (v/v) acetonitrile 30–55 min, linear gradient 13%–22% (v/v) acetonitrile 55–70 min, linear gradient 22%–40% (v/v) acetonitrile

(B)	Merck Purospher STAR C_18_ column	0–30 min, isocratic 13% (v/v) acetonitrile 30–55 min, linear gradient 13%–22% (v/v) acetonitrile 55–70 min, linear gradient 22%–40% (v/v) acetonitrile

(C)	Merck Purospher STAR C_18_ column	0–30 min, isocratic 18% (v/v) acetonitrile 30–60 min, linear gradient 18%–30% (v/v) acetonitrile 60–70 min, isocratic 30% (v/v) acetonitrile

**Table 2:** The different ultrasonic bath extraction conditions (see Figure 3).

Number	Individual experiments	Extraction conditions
(1)	Effect of extraction solvent	Methanol concentration (30%, 50%, 70%, and 100% aqueous methanol), solvent volume 100 mL, and extraction time 30 min

(2)	Effect of ultrasonic time	Extraction time (10, 20, 30, and 40 min), extraction solvent 70% aqueous methanol, and the ratio sample/extraction solvent 3/100 (g/mL)

(3)	Effect of solvent volumes	The ratio sample/extraction solvent (3/50, 3/100, and 3/150 g/mL), extraction solvent 70% aqueous methanol, and extraction time 30 min

**Table 3:** Regression data, LODs, and LOQs for the six active constituents.

Analytes	Regression equation (*Y* = *aX* + *b*)	*R* ^2^	Linear range (*μ*g/mL)	LOD (*μ*g/mL)	LOQ (*μ*g/mL)
LLD	*Y* = 2.2201 × 10^4^ *X* + 10.2083 × 10^2^	0.9993	0.3184~31.84	0.3184	0.9652
AGD	*Y* = 1.4863 × 10^4^ *X* − 1.2973 × 10^3^	0.9991	0.1165~34.95	0.1165	0.3599
RA	*Y* = 2.9184 × 10^4^ *X* + 2.2098 × 10^3^	0.9994	0.2184~21.84	0.2184	0.6665
DTD	*Y* = 1.4021 × 10^4^ *X* − 6.8541 × 10^2^	0.9994	0.2784~27.84	0.2784	0.8501
TLN	*Y* = 3.4167 × 10^4^ *X* − 1.9921 × 10^4^	0.9987	0.3800~39.50	0.3800	1.1508
ATD	*Y* = 5.7370 × 10^3^ *X* + 3.5575 × 10^2^	0.9989	0.2340~70.20	0.2340	0.7111

*Y*: peak area; *X*: concentration (*μ*g/mL).

**Table 4:** Precision for the six active constituents (mean ± SD, *n* = 6).

Analytes	Precision intraday		Precision interday	
	Content (%)	RSD (%)	Content (%)	RSD (%)
LLD	0.4112 ± 0.0043	1.05	0.4126 ± 0.0046	1.11
AGD	0.1509 ± 0.0019	1.23	0.1519 ± 0.0027	1.75
RA	0.4731 ± 0.0074	1.56	0.4721 ± 0.0068	1.44
DTD	0.5358 ± 0.0052	0.97	0.5377 ± 0.0065	1.21
TLN	0.6566 ± 0.0049	0.75	0.6549 ± 0.0058	0.88
ATD	0.6303 ± 0.0083	1.32	0.6315 ± 0.0099	1.57

**Table 5:** Repeatability and stability for the investigated analytes.

Analytes	Repeatability (mean ± SD, *n* = 6)		Stability (12 h) (mean ± SD, *n* = 7)	
	Content (%)	RSD (%)	Content (%)	RSD (%)
LLD	0.4155 ± 0.0056	1.35	0.4147 ± 0.0078	1.87
AGD	0.1571 ± 0.0019	1.21	0.1561 ± 0.0010	0.61
RA	0.4752 ± 0.0073	1.53	0.4712 ± 0.0072	1.53
DTD	0.5388 ± 0.0097	1.80	0.5317 ± 0.0094	1.76
TLN	0.6517 ± 0.0076	1.16	0.6561 ± 0.0090	1.37
ATD	0.6336 ± 0.0070	1.11	0.6329 ± 0.0070	1.11

**Table 6:** Recovery test for the determination of the investigated analytes (mean ± SD, *n* = 3).

Analytes	Originals (mg)	Spiked (mg)	Found (mg)	Recovery (%)	RSD (%)
LLD	0.6209	0.3184	0.9344 ± 0.0036	98.43 ± 1.10	1.12
		0.6368	1.2483 ± 0.0047	98.53 ± 0.72	0.73
		0.9552	1.5667 ± 0.0101	99.00 ± 1.05	1.06

AGD	0.2347	0.1165	0.3508 ± 0.0021	99.63 ± 1.80	1.81
		0.2330	0.4678 ± 0.0033	100.06 ± 1.44	1.44
		0.3495	0.5849 ± 0.0037	100.20 ± 1.05	1.05

RA	0.7057	0.3276	1.0280 ± 0.0033	98.33 ± 1.01	1.02
		0.6552	1.3565 ± 0.0106	99.34 ± 1.59	1.60
		0.9828	1.6867 ± 0.0056	99.80 ± 0.59	0.59

DTD	0.8085	0.4176	1.2258 ± 0.0016	99.88 ± 0.41	0.41
		0.8352	1.6528 ± 0.0085	101.09 ± 1.02	1.01
		1.2528	2.0573 ± 0.0123	99.66 ± 1.00	1.00

TLN	0.9779	0.3950	1.3696 ± 0.0009	99.10 ± 0.19	0.19
		0.9875	1.9715 ± 0.0071	100.63 ± 0.75	0.74
		1.5800	2.5592 ± 0.0059	100.06 ± 0.38	0.38

ATD	0.9535	0.4680	1.4236 ± 0.0073	100.41 ± 1.53	1.52
		0.9360	1.8888 ± 0.0168	99.93 ± 1.79	1.79
		1.4040	2.3670 ± 0.0166	100.66 ± 1.20	1.19

**Table 7:** Contents of the six analytes in Yixin Badiranjibuya Granules from different manufacturers in China (mean ± SD, *n* = 3).

Number	Analytes (mg/g)					
	LTD	AGG	RA	DSD	TLN	ACG
1	0.4135 ± 0.0065	0.1561 ± 0.0044	0.4756 ± 0.0078	0.5299 ± 0.0078	0.6562 ± 0.0055	0.6313 ± 0.0066
2	0.4101 ± 0.0071	0.1501 ± 0.0058	0.4721 ± 0.0078	0.5367 ± 0.0062	0.6570 ± 0.0047	0.6297 ± 0.0040
3	0.4164 ± 0.0056	0.1536 ± 0.0039	0.4772 ± 0.0081	0.5319 ± 0.0054	0.6465 ± 0.0050	0.6275 ± 0.0046
4	0.3256 ± 0.0034	0.1211 ± 0.0063	0.4188 ± 0.0065	0.3522 ± 0.0037	0.5899 ± 0.0038	0.6066 ± 0.0072
5	0.3233 ± 0.0053	0.1135 ± 0.0045	0.4012 ± 0.0047	0.3556 ± 0.0028	0.5855 ± 0.0087	0.6012 ± 0.0057
6	0.3185 ± 0.0068	0.1298 ± 0.0071	0.4109 ± 0.0059	0.3601 ± 0.0035	0.5923 ± 0.0067	0.6089 ± 0.0044
7	0.3245 ± 0.0072	0.1166 ± 0.0049	0.4233 ± 0.0076	0.2611 ± 0.0032	0.5211 ± 0.0075	0.5344 ± 0.0069
7	0.1109 ± 0.0031	0.0698 ± 0.0032	0.4212 ± 0.0064	0.1623 ± 0.0017	0.3256 ± 0.0055	0.2516 ± 0.0066
9	0.1082 ± 0.0042	0.0523 ± 0.0052	0.4203 ± 0.0056	0.1588 ± 0.0028	0.3234 ± 0.0049	0.2477 ± 0.0053
10	0.3399 ± 0.0059	0.1377 ± 0.0078	0.4256 ± 0.0071	0.3736 ± 0.0067	0.6213 ± 0.0058	0.5555 ± 0.0028
11	0.3265 ± 0.0038	0.1398 ± 0.0058	0.4311 ± 0.0063	0.3677 ± 0.0034	0.6139 ± 0.0067	0.5333 ± 0.0034

## Appendix

See Figure 5.

*NMR Data of Tilianin, Diosmetin-7-O-β-D-glucuronide, and Acacetin-7-O-β-D-glucuronide*. Tilianin (TLN) [23] was isolated as amorphous pale-yellow powder. Its molecular formula C_22_H_20_O_10_ was determined by ESI-MS at *m*/*z* 445 [M-H]^−^.

^1^H-NMR (DMSO-d_6_, 400 MHz) *δ*: 12.86 (1 H,* s*, OH-5), 8.04 (2 H,* d*,* J *= 8.8 Hz, H-2′, 6′), 7.12 (2 H,* d*,* J* = 8.8 Hz, H-3′, 5′), 6.90 (1 H,* s*, H-3), 6.83 (1 H,* s*, H-8), 6.44 (1 H,* s*, H-6), 5.05 (1 H,* d*,* J* = 7.6, H-1′′), 3.86 (3 H,* s*, –OCH_3_).

^13^C-NMR (DMSO-d_6_, 150 MHz) *δ*: 164.0 (C-2), 104.0 (C-3), 182.1 (C-4), 161.2 (C-5), 99.8 (C-6), 163.2 (C-7), 95.1 (C-8), 157.1 (C-9), 105.3 (C-10), 122.9 (C-1′), 128.6 (C-2′), 114.8 (C-3′), 162.6 (C-4′), 114.8 (C-5′), 128.6 (C-6′), 100.2 (C-1′′), 73.3 (C-2′′), 77.3 (C-3′′), 69.8 (C-4′′), 76.6 (C-5′′), 60.8 (C-6′′), 55.7 (–OCH_3_).

Diosmetin-7-*O*-*β*-D-glucuronide (DSG) [24] was isolated as amorphous yellow powder. Its molecular formula C_22_H_20_O_12_ was determined by ESI-MS at *m*/*z* 475 [M-H]^−^.

^1^H-NMR (DMSO-d_6_, 400 MHz) *δ*: 12.89 (1 H,* s*, OH-5), 7.53 (1 H,* d*,* J *= 8.4 Hz, H-6′), 7.46 (1 H,* s*, H-2′), 7.08 (1 H,* d*,* J* = 8.4 Hz, H-5′), 6.79 (1 H,* s*, H-8), 6.77 (1 H,* s*, H-8), 6.43 (1 H,* s*, H-6), 5.12 (1 H,* d*,* J* = 6.8, H-1′′), 5.14 (1 H,* s*, H-1′′′), 3.86 (3 H,* s*, –OCH_3_).

^13^C-NMR (DMSO-d_6_, 150 MHz) *δ*: 164.3 (C-2), 104.0 (C-3), 182.1 (C-4), 161.4 (C-5), 99.9 (C-6), 163.2 (C-7), 95.0 (C-8), 157.2 (C-9), 105.7 (C-10), 123.1 (C-1′), 113.4 (C-2′), 147.2 (C-3′), 151.6 (C-4′), 112.5 (C-5′), 119.0 (C-6′), 99.9 (C-1′′), 73.2 (C-2′′), 74.7 (C-3′′), 72.0 (C-4′′), 76.5 (C-5′′), 172.6 (C-6′′), 56.0 (–OCH_3_).

Acacetin-7-*O*-*β*-D-glucuronide (ACG) [25] was isolated as amorphous pale-yellow powder. Its molecular formula C_22_H_20_O_11_ was determined by ESI-MS at *m*/*z* 459 [M-H]^−^.

^1^H-NMR (DMSO-d_6_, 400 MHz) *δ*: 12.86 (1 H,* s*, OH-5), 8.03 (2 H,* d*,* J *= 8.0 Hz, H-2′, 6′), 7.12 (2 H,* d*,* J* = 8.0 Hz, H-3′, 5′), 6.89 (1 H,* s*, H-3), 6.84 (1 H,* s*, H-8), 6.45 (1 H,* s*, H-6), 5.12 (1 H,* brs*, H-1′′), 3.87 (3 H,* s*, –OCH_3_).

^13^C-NMR (DMSO-d_6_, 150 MHz) *δ*: 164.0 (C-2), 103.9 (C-3), 182.1 (C-4), 161.3 (C-5), 99.8 (C-6), 163.2 (C-7), 95.0 (C-8), 157.1 (C-9), 105.6 (C-10), 122.8 (C-1′), 128.5 (C-2′), 114.7 (C-3′), 162.6 (C-4′), 114.7 (C-5′), 128.5 (C-6′), 99.8 (C-1′′), 73.1 (C-2′′), 76.4 (C-3′′), 72.0 (C-4′′), 74.6 (C-5′′), 171.1 (C-6′′), 55.6 (–OCH_3_).
